# *Neisseria gonorrhea* in Ethiopia, prevalence among STI suspected patients and its antimicrobial susceptibility: a systematic review and meta-analysis

**DOI:** 10.3389/fmicb.2024.1390001

**Published:** 2024-04-12

**Authors:** Mengistie Yirsaw Gobezie, Nuhamin Alemayehu Tesfaye, Tewodros Solomon, Mulat Belete Demessie, Tesfaye Dessale Kassa, Teklehaimanot Fentie Wendie, Ermiyas Alemayehu, Minimize Hassen

**Affiliations:** ^1^Department of Clinical Pharmacy, School of Pharmacy, College of Medicine and Health Sciences, Wollo University, Dessie, Ethiopia; ^2^Department of Clinical Pharmacy, College of Health Sciences, Mekelle University, Mekelle, Ethiopia; ^3^Department of Medical Laboratory Sciences, College of Medicine and Health Sciences, Wollo University, Dessie, Ethiopia

**Keywords:** *Neisseria gonorrhea*, prevalence, antimicrobial susceptibility, systematic review, meta-analysis, sexually transmitted infections, Ethiopia

## Abstract

**Introduction:**

*Neisseria gonorrhea* (*N. gonorrhea*) represents a significant causative agent of sexually transmitted infections (STIs), posing considerable global health challenges. Despite the presence of diagnostic tools and empirically guided therapies, the escalating AMR of *N. gonorrhea* continues to pose a threat. This study aims to assess the prevalence of *N. gonorrhea* among STI suspected patients in Ethiopia and explore the patterns of AMR to common antimicrobials.

**Methods:**

Following the Preferred Reporting Items for Systematic Reviews and Meta-Analyses (PRISMA) guidelines, we conducted a systematic review and meta-analysis. A thorough search of electronic databases from July 11 to July 24, 2023, identified 10 eligible studies. Data were extracted and analyzed using a random-effects model. Heterogeneity was assessed using the I^2^ statistic, and publication bias was evaluated through Egger’s regression test and funnel plots.

**Results:**

The overall pooled prevalence of *N. gonorrhea* among STI suspected patients in Ethiopia was 20% (95% confidence interval (CI): 8–30, I^2^ = 99.0%; *p*-value <0.001). Substantial regional variations were observed, with the highest prevalence in Addis Ababa (55, 95% CI: 45–65) and the lowest in the Southern Nations, Nationalities, and Peoples’ Region (SNNPR) (4, 95% CI: 2–8). The pooled prevalence of AMR to ciprofloxacin, ceftriaxone, azithromycin, benzylpenicillin, tetracycline, and spectinomycin was 37, 9, 10, 79, 93, and 2%, respectively. Significant heterogeneity existed between studies (I^2^ = 99.0%; *p* value <0.001). Publication bias, identified through funnel plot examination and Egger’s regression test (*p* < 0.001), execution of trim and fill analysis resulted in an adjusted pooled prevalence of (6.2, 95% CI: −6.8 to 19.3).

**Conclusion:**

The prevalence of *N. gonorrhea* among STI suspected patients in Ethiopia is alarming, particularly in specific regions. The elevated AMR to ciprofloxacin underscores the immediate need for alternative treatment options and enhanced surveillance systems. Future initiatives should prioritize strengthening laboratory capacities and implementing targeted interventions to curtail *N. gonorrhea* transmission and prevent the emergence of AMR.

**Systematic Review Registration:**

https://www.crd.york.ac.uk/prospero, identifier CRD42023459698.

## Introduction

*Neisseria gonorrhea* is a Gram-negative diplococcus bacterium that causes one of the most prominent (STIs called gonorrhea; Whittles et al., 2018; Yakobi and Pooe, 2022). Worldwide, about 87 million incident gonorrhea cases happened among persons aged 15–49 years with an incidence rate of 26/1,000 men. The same evidence spotlighted that, in 2016, roughly more than 1 million STIs were acquired daily globally with 30 million documented cases of *N. gonorrhea* (Rowley et al., 2019). Besides, *N. gonorrhea* is known to cause male urethritis, female endocervicitis, and fatal reproductive complications such as infertility, miscarriage, ectopic pregnancy, pelvic inflammatory disease, and epididymitis that will eventually lead to substantial morbidity and mortality (Cohen, 2012).

In most of the developing countries, including Ethiopia, STI treatment is based on a syndromic management approach. This is mainly due to the shortage of laboratory equipment and resources in primary health care facilities which serves as an initial point of contact for entertaining STI cases (Feglo and Opoku, 2014). Empirical antibiotic therapy remains the gold standard treatment for *N. gonorrhea* infections owing to the lack of appropriate laboratory facilities for undergoing advanced microbiological tests like culture and sensitivity testing, and the higher cost of laboratory services (Acheampong et al., 2018). Although the syndromic approach has well-established financial merits, it is also questioned for its enormous contribution to the emergence of AMR (Ventola, 2015).

Recently, the progressive development and spread of *N. gonorrhea* AMR pose a significant sexual health threat across the globe (Whiley et al., 2012; Unemo and Shafer, 2014). Because of the widespread resistance to potent antibiotics such as sulfonamides, penicillin, tetracycline, and quinolones, the recent therapeutic alternatives for *N. gonorrhea* infections have been severely hampered (Olsen et al., 2013; Lebedzeu et al., 2015). A nascent study reported a prodigious degree of *N. gonorrhea* resistance to commonly used antibiotics in Sub-Saharan countries (Tadesse et al., 2017), while globally, new and untreated cases of *N. gonorrhea* infections are astoundingly rising due to treatment failures and the disease’s asymptomatic nature (Lovett and Duncan, 2019; Springer and Salen, 2023). Consequently, to stem down infection rate, various concerned stakeholders including the World Health Organization (WHO), the United States of America Centers for Disease Control and Prevention (USA-CDC), and other regulatory bodies have designed global action plans (World Health Organization, 2012). The WHO advocated that national *N. gonorrhea* AMR surveillance should be conducted at regular intervals and treatment modification must be implemented if the observed resistance prevalence is above 5% for any antibiotics (World Health Organization, 2012; Unemo and Shafer, 2014). However, this stringent recommendation is not affordable to many developing countries.

Therefore, this systematic review and meta-analysis aim to synthesize the available evidence on the prevalence of *N. gonorrhea* among STI suspected patients in Ethiopia and assess its antimicrobial susceptibility profile. By consolidating this information, we seek to provide insights that can inform policy-makers, healthcare providers, and researchers in Ethiopia and beyond, toward the development of effective strategies for *N. gonorrhea* control and management in resource-limited settings.

## Methods

### Reporting

The study adhered to the Preferred Reporting Items for Systematic Reviews and Meta-analysis (PRISMA) (Liberati et al., 2009) guideline for reporting. Furthermore, the protocol for this systematic review and meta-analysis has been registered in the Prospero database under the registration number PROSPERO 2023: CRD42023459698.

### Databases and search strategy

A comprehensive search was conducted in electronic databases including PubMed, Google Scholar, Hinari, SCOPUS, and EMBASE from July 11–24, 2023. The search strategy was collaboratively developed by two study authors, and its execution was carried out by another two individuals from June 20 to July 10, 2023. The search utilized specific terms such as *Neisseria gonorrhea*, *N. gonorrhea*, *Micrococcus gonorrhea*, *Gonococcus*, Sexually transmitted infection, sexually transmitted disease, STI, STD, and Ethiopia. Boolean operators “AND” and “OR” were employed in the search strings.

### Inclusion and exclusion criteria

The search encompassed studies published from January 1, 2010, to July 11, 2023. Inclusion criteria comprised studies meeting the following conditions: (I) investigations involving suspected STI patients or reporting antimicrobial susceptibility of *Neisseria gonorrhea*; (II) observational studies, encompassing cross-sectional studies, cohorts, randomized controlled trials, or surveillance designs; (III) studies conducted in Ethiopia; (IV) studies published in the English language. Exclusion criteria encompassed case reports, case–control studies, reviews, commentaries, and editorials. Additionally, conference abstracts were not included in the search.

### Study selection and quality assessment

We employed Endnote version 20.5 (Gotschall, 2021). Reference Manager to eliminate duplicated studies. The titles and abstracts were independently screened by two authors (MY and TD) to determine which articles should undergo a full-text review. The full text of the remaining articles was then obtained, and two investigators, EA and MH, independently assessed them for eligibility. The quality of the studies was evaluated using the JBI critical appraisal checklist for studies reporting prevalence data (Institute JB, 2017). The following criteria were utilized for appraising the selected studies: (I) Appropriateness of the sampling frame for addressing the target population, (II) Appropriateness of the study participants’ sampling technique and adequacy of the sample size, (III) Detailed description of study subjects and setting, (IV) Sufficient analysis of the data and validity reliability of methods used for measuring AMR and the prevalence of *N. gonorrhea*, (V) Appropriateness of the statistical analysis used and adequacy of the sample size. Disagreements were resolved through consensus. Studies scoring five and above out of nine were considered to have a low risk.

### Data extraction

Data extraction was conducted by two authors (MY and MH) following the established data extraction format. Whenever discrepancies arose, the procedure was repeated to ensure accuracy. The identified articles that met our inclusion criteria were then compiled into tables by TD, TS, and NA. These tables included information on authors, study period, publication year, study design, study setting, study population, study region, sample size, number of *N. gonorrhea* isolates, AMR prevalence for tested antimicrobials, and the antimicrobial sensitivity test method. It’s important to note that not all studies provided information on AMR or prevalence.

### Outcome of interest

The study aimed to investigate two primary outcomes concerning *N. gonorrhea* among individuals suspected of STI in Ethiopia. Firstly, the study focused on determining the prevalence of *N. gonorrhea*. This was calculated by dividing the number of *N. gonorrhea*-positive individuals by the total number of individuals tested, with each prevalence data point pooled to estimate its overall prevalence. Secondly, the study examined the antimicrobial resistance profiles of *N. gonorrhea* against specific antibiotics. To assess the pooled prevalence of antimicrobial resistance for *N. gonorrhea* for selected antimicrobials, all included primary studies for this meta-analysis adhered to the Clinical and Laboratory Standards Institute (CLSI) guidelines as a standardized approach. The selected antibiotics for testing discs in the included primary studies were penicillin (10 IU), tetracycline (30 μg), ciprofloxacin (5 μg), ceftriaxone (30 μg), spectinomycin (100 μg), and azithromycin (15 μg).

### Data analysis

To estimate the prevalence of *N. gonorrhea* among suspected STI patients and its AMR prevalence for tested antimicrobials, we employed a weighted inverse variance random-effects model (DerSimonian and Kacker, 2007). Subgroup analysis based on the region where the studies were conducted was conducted to adjust for variation in pooled prevalence estimates. Heterogeneity among studies was evaluated using a forest plot, meta-regression, and the I^2^ statistic, where values of 25, 50, and 75% represented low, moderate, and high heterogeneity, respectively (Higgins et al., 2003). The significance of heterogeneity was assessed through a Q test, with a *p*-value less than 0.05 indicating significance.

The findings were visually presented using a forest plot. To assess publication bias, a Funnel plot, and Egger’s regression test were employed, with a *p*-value less than 0.05 in Egger’s test suggesting significant publication bias. Additionally, Trim and fill analysis were applied to check for publication bias (Peters et al., 2006). A sensitivity analysis was conducted to ensure the stability of the summary estimate. The meta-analysis was performed using STATA version 17 (StataCorp, 2023) Statistical software.

## Results

### Characteristics of included studies

**Figure 1:**
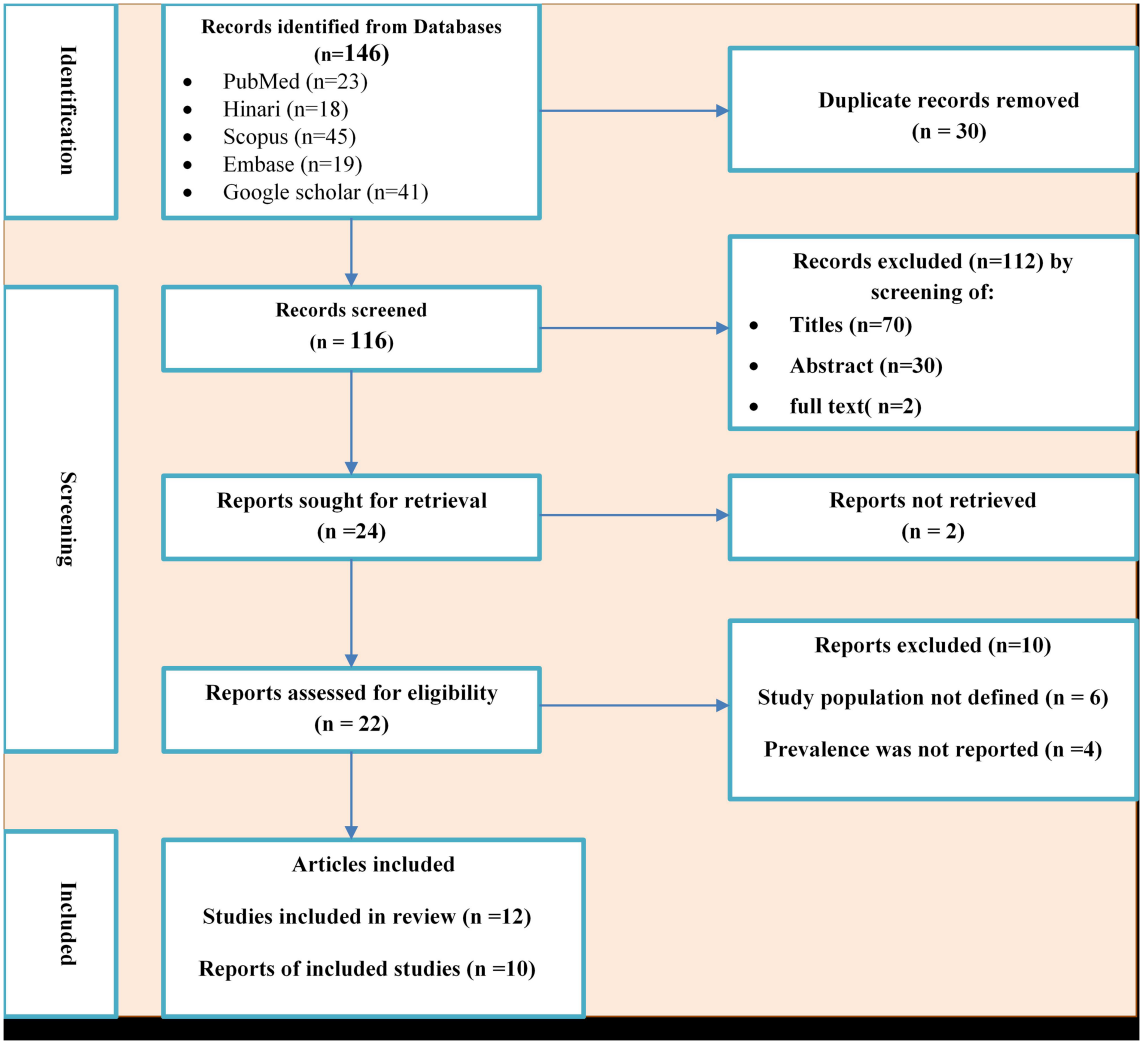
PRISMA flow diagram of the included studies for the systematic review and meta-analysis of the prevalence of *Neisseria gonorrhea* among STI suspected patients in Ethiopia.

A total of 155 potential studies were identified, comprising 23 articles from PubMed, 18 from Hinari (research4life), 19 from EMBASE, 45 from Scopus, and 50 from various other sources. The outcomes of the search and the reasons for exclusion during the study selection process are depicted in Figure 1. Ultimately, 10 articles were included to evaluate the prevalence of *N. gonorrhea* among suspected STI patients in Ethiopia. All the included studies adopted a cross-sectional study design, with three of them specifically conducted in the Amhara region (Tibebu et al., 2013; Mulu et al., 2015; Geremew et al., 2017; Yeshanew and Geremew, 2018), one in Oromia (Sahile et al., 2020), two in SNNPR (Hailemariam et al., 2013; Tadele et al., 2019; Zenebe et al., 2021), two in Addis Ababa (Fentaw et al., 2020; Ayalew et al., 2022), one in Gambella (Ali et al., 2016) and one in Tigray (Kahsay et al., 2023). The prevalence of antimicrobial-resistant *N. gonorrhea* for specific antibacterial agents was reported in nine studies. The study encompassed a total of 3,558 participants, including 2,799 individuals suspected of having STIs and 759 asymptomatic individuals who participated in the included studies. Table 1 provides an overview of the characteristics of the included studies.

**Table 1:** Characteristics of studies included for the systematic review and meta-analysis of the prevalence of *N. gonorrhea* among STI suspected patients in Ethiopia.

Authors	Study period	Year of publication	Regions	Study design	Study setting	Study population	Sample size	NNG isolates	Standards used	AST employed	Risk of bias
Hailemariam et al.	2010–11	2013	SNNPR	CS	Hospitals	Suspected STIs	215	11	CLSI	DDM	Low
Tibebu et al.	2006–12	2013	Amhara	RS	Health facilities	Suspected STIs	352	29	CLSI	DDM	Low
Mulu et al.	2013	2015	Amhara	CS	Hospitals	Asymptomatic	409	4	CLSI	DDM	Low
Ali et al.	2015	2016	Gambella	CS	Hospitals	Suspected STIs	186	21	CLSI	DDM	Low
Geremew et al.	2014	2017	Amhara	CS	Hospitals HC	Suspected STIs	120	25	NA	NA	Low
Yeshanew et al.	2016	2018	Amhara	CS	Hospitals HC	Suspected STIs	120	25	CLSI	DDM	Low
Tadele et al.	2017	2019	SNNPR	CS	Clinics	Suspected STIs	338	11	NA	NA	Low
Fentaw et al.	2013–14	2020	Addis Ababa	CS	HC	Suspected STIs	599	361	CLSI	DDM	Low
Sahile et al.		2020	Oromia	CS	Clinics	Suspected STIs	315	31	CLSI	DDM	Low
Zenebe et al.	2020	2021	SNNPR	CS	Hospitals	Asymptomatic	350	15	NA	NA	Low
Ayalew et al.	2019–20	2022	Addis Ababa	CS	HC	Suspected STIs	325	163	NA	NA	Low
Kahsay et al.	2018	2023	Tigray	CS	Clinics	Suspected STIs	229	23	CLSI	DDM	Low

CS, cross sectional; SNNPR, southern nation nationalities and people region; HC, health center; NNG, Number of *N. gonorrhea*; CLSI, Clinical and Laboratory Standards Institute; AST, Antimicrobial Susceptibility Test; NA, Not applicable; DDM, Disc Diffusion Method.

### Quality of the included studies

All studies underwent evaluation using the JBI critical appraisal checklist for studies reporting prevalence data. The assessments utilizing the JBI quality appraisal checklists revealed that none of the included studies were deemed to be of poor quality and, therefore, none were excluded from the meta-analysis.

### Meta-analysis

#### Pooled prevalence of *Neisseria gonorrhea* among STI suspected patients in Ethiopia

**Figure 2:**
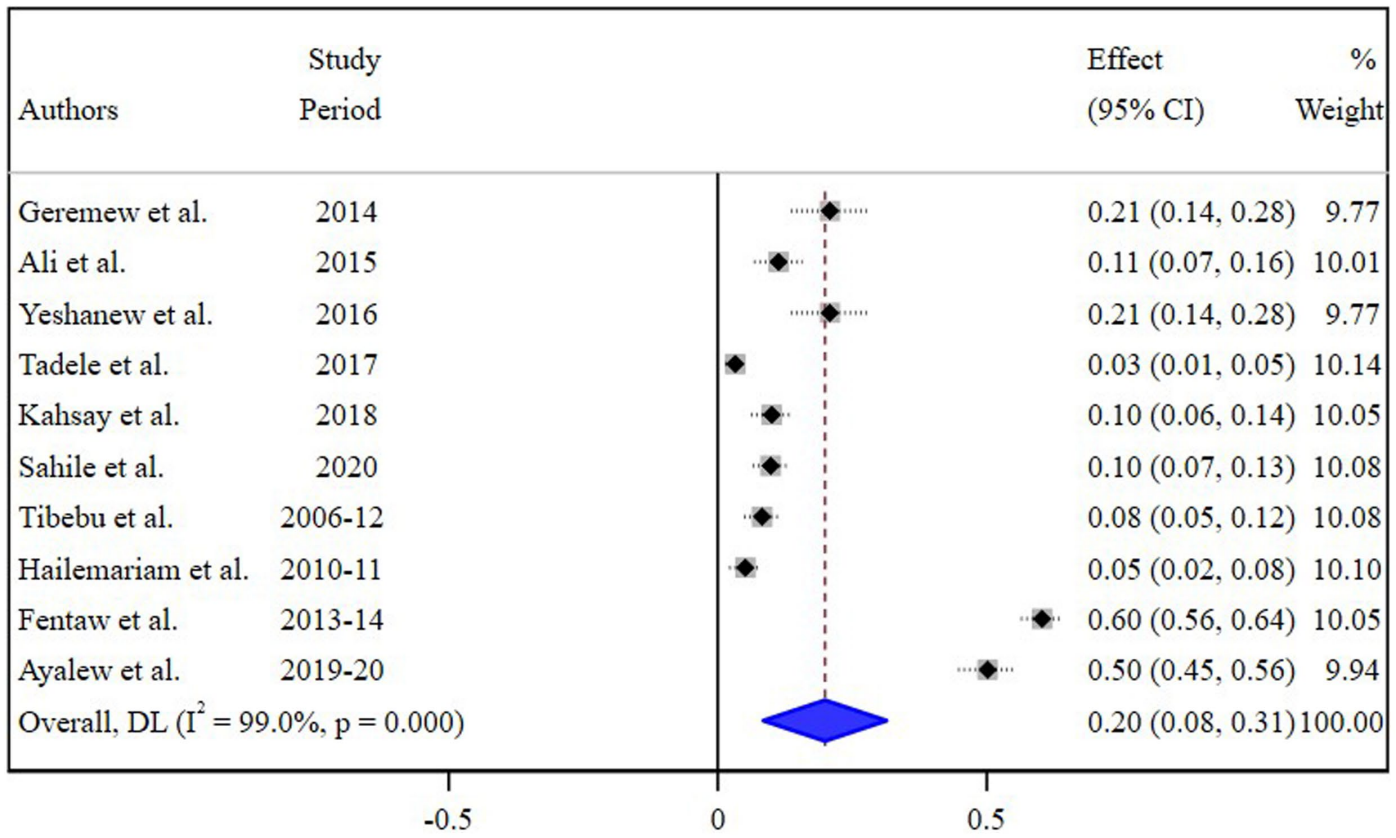
Pooled estimate of the prevalence of *N. gonorrhea* among STI suspected patients in Ethiopia.

The estimated pooled prevalence of *N. gonorrhea* among STI suspected patients in Ethiopia was 20% [95% Confidence Interval (CI) 8–30, I^2^ = 99.0%; *p* < 0.001] (Figure 2).

### Subgroup analysis

**Figure 3:**
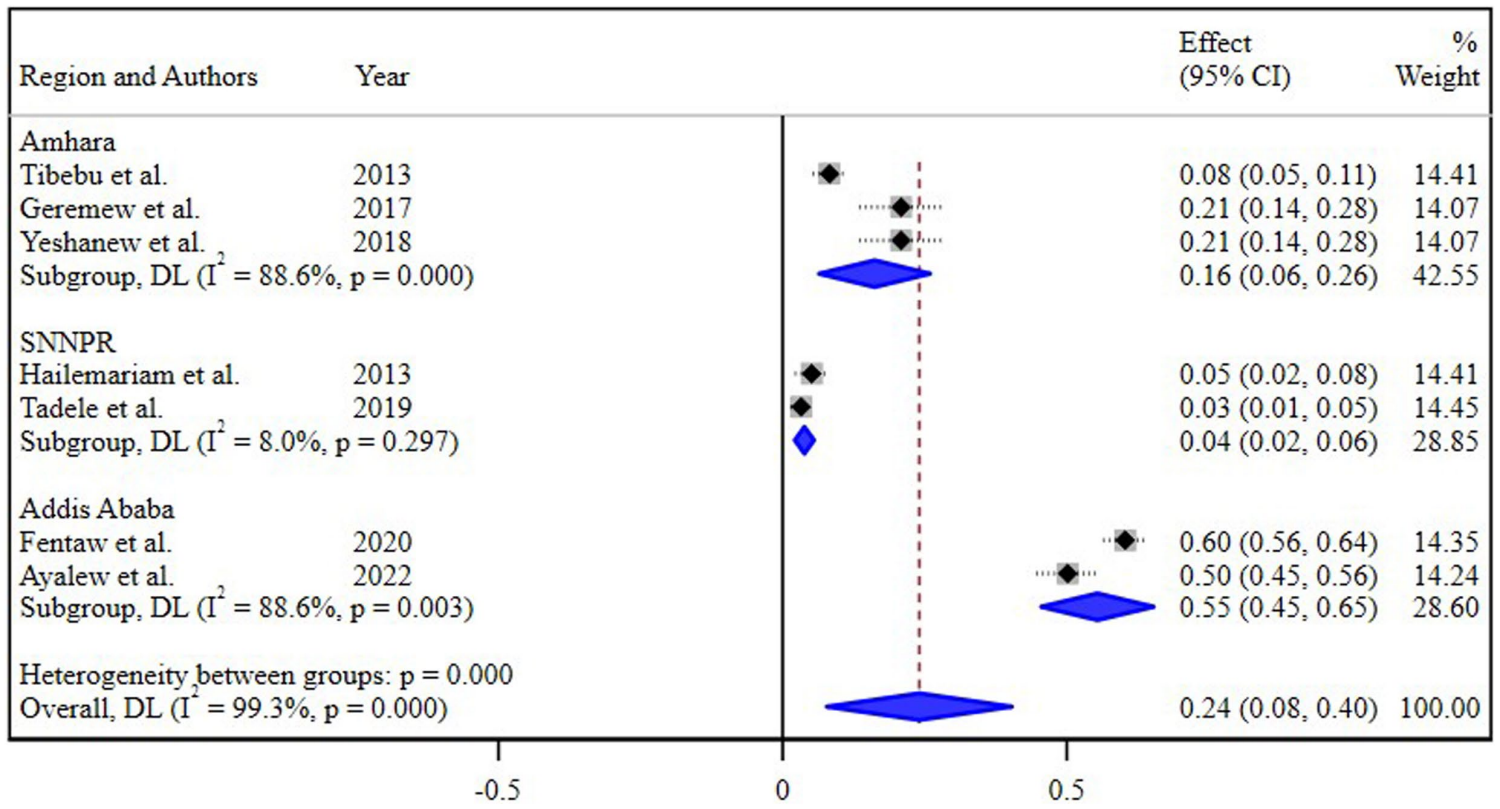
Subgroup analysis of the prevalence of *N. gonorrhea* by regions.

**Figure 4:**
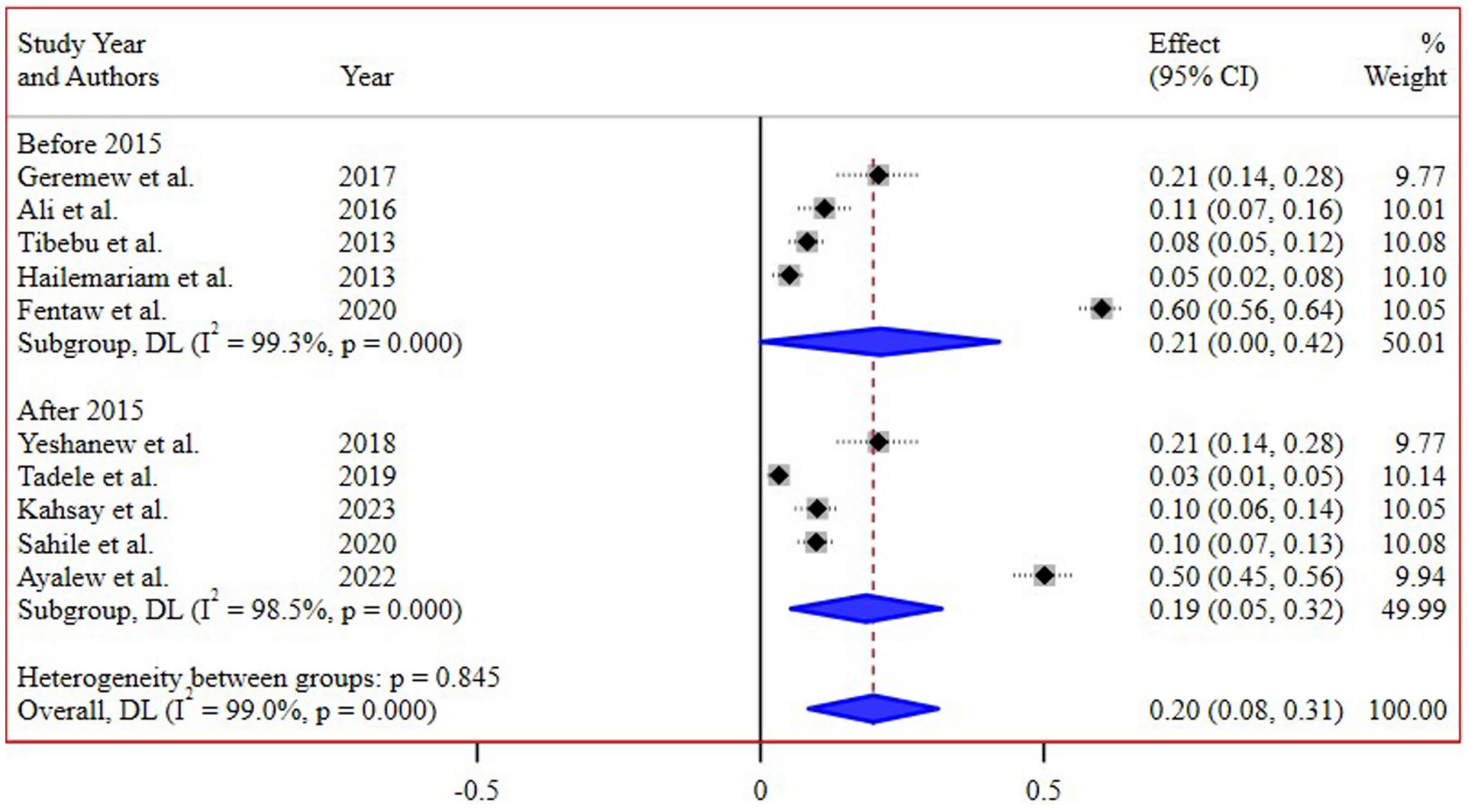
Subgroup analysis of the prevalence of *N. gonorrhea* by study periods.

The subgroup analysis based on different regions of Ethiopia revealed the highest pooled prevalence of 55% (95% CI 45, 65) in Addis Ababa, followed by 16% (95% CI 8, 26) in the Amhara region. Conversely, the lowest prevalence of 4% (95% CI 2, 8) was reported in the Southern Nations, Nationalities, and Peoples’ Region (SNNPR) (Figure 3). Additionally, a subgroup analysis conducted based on the study periods did not demonstrate a significant difference in the pooled prevalence of *N. gonorrhea* among the groups (Figure 4).

### Heterogeneity analysis

**Figure 5:**
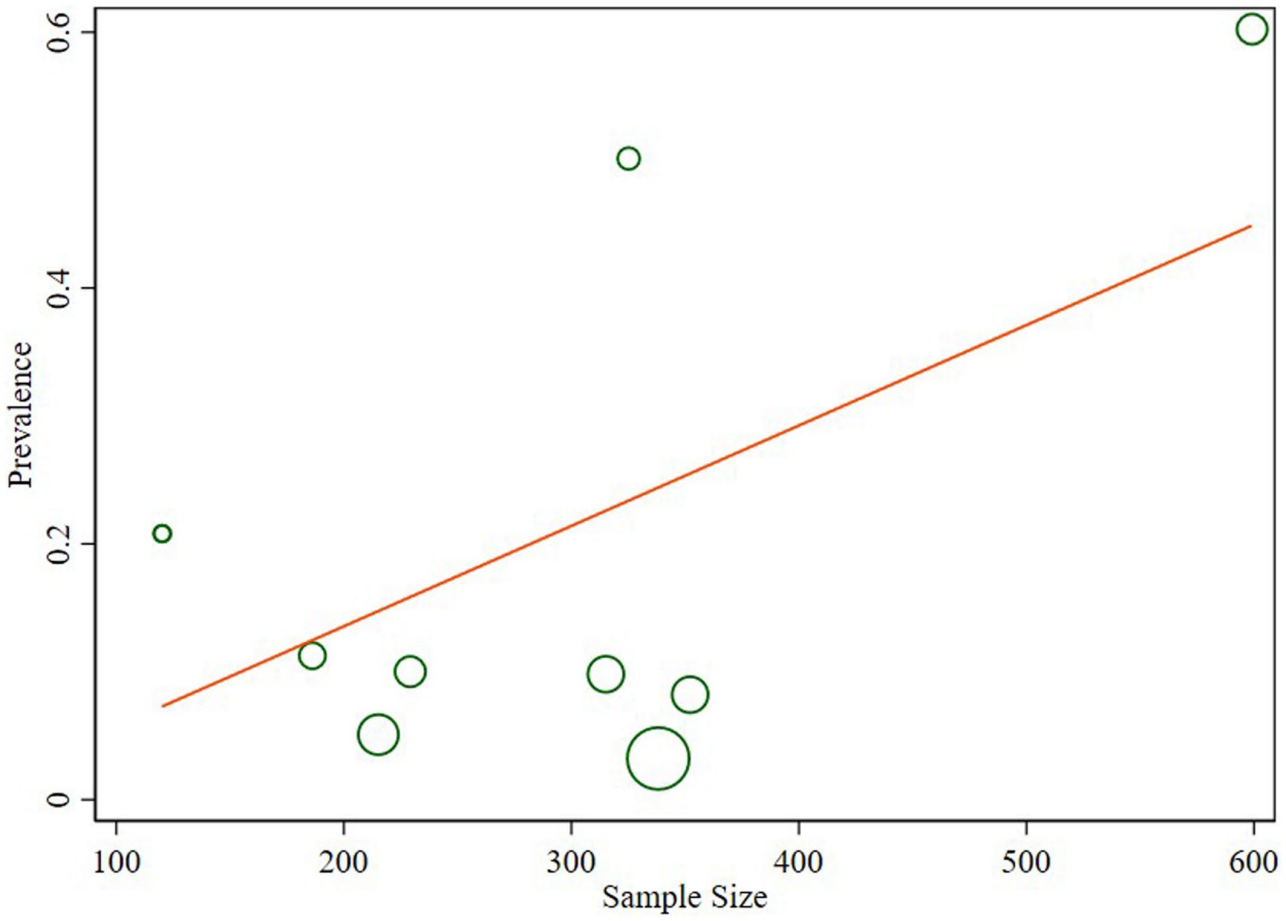
Meta regression of the prevalence of *N. gonorrhea* and sample size.

The studies included in the analysis exhibited substantial heterogeneity (I^2^ = 98.1%; *p* < 0.001), which was not adequately addressed by a weighted inverse variance random-effects model. To further explore this heterogeneity, we employed a forest plot for a subjective assessment and carried out a subgroup analysis. Ultimately, meta-regression was conducted using sample size, revealing the absence of significant heterogeneity (Table 2; Figure 5).

**Table 2:** Meta regression of prevalence of *N. gonorrhea* and sample size.

*P*	Coefficient	Std. err.	*t*	*P* > |*t*|	[95% CI]	
Sample size	0.0007862	0.0004043	1.94	0.088	−0.0001462	0.0017186
_cons	−0.0215076	0.1258618	−0.17	0.869	−0.3117454	0.2687301

### Publication bias

**Figure 6:**
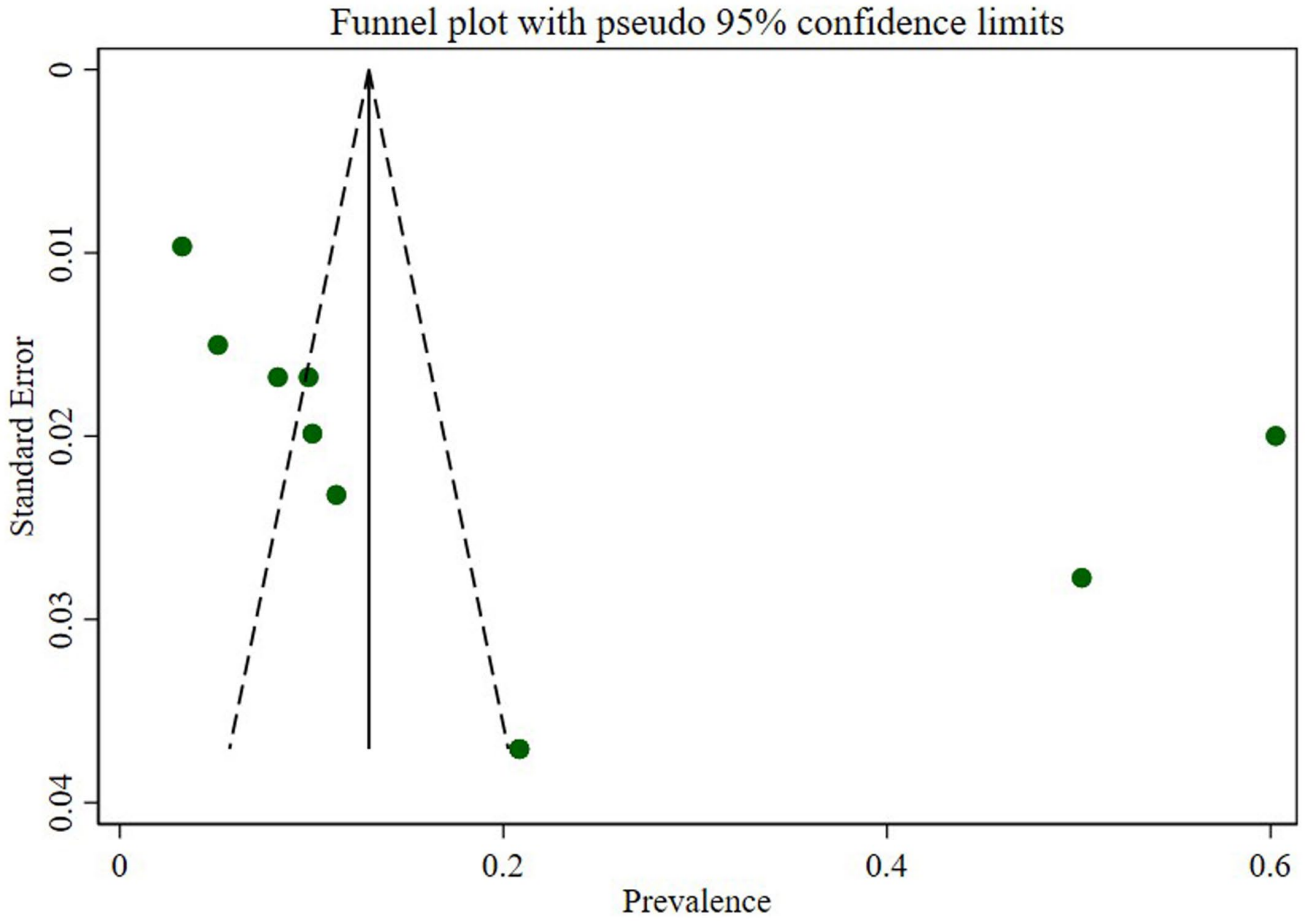
Funnel plot of prevalence *N. gonorrhea* among STI suspected patients in Ethiopia.

We assessed publication bias through a subjective examination of the funnel plot and conducted Egger’s regression test, yielding a *p*-value of 0.001, indicating the presence of publication bias. Subsequently, we performed a trim and fill analysis, which added five studies, suggesting the existence of overlooked small studies (Figure 6).

### Sensitivity analysis

We conducted a sensitivity analysis for the prevalence of *N. gonorrhea* using a random effects model (Table 3). The exclusion of each study did not significantly affect the prevalence of *N. gonorrhea* among STI suspected patients in Ethiopia.

**Table 3:** Sensitivity analysis of studies included for estimation of pooled prevalence of *N. gonorrhea* among STI suspected patients in Ethiopia.

Study omitted	Estimate	[95% CI]	
Geremew et al. (2017)	0.19847949	0.07066959	0.32628939
Ali et al. (2016)	0.20910977	0.0773275	0.34089205
Yeshanew and Geremew (2018)	0.19847949	0.07066959	0.32628939
Tadele et al. (2019)	0.21830221	0.08058105	0.35602334
Sahile et al. (2020)	0.21082845	0.07387608	0.34778082
Ayalew et al. (2022)	0.16595517	0.05014473	0.28176561
Tibebu et al. (2013)	0.21241057	0.08264342	0.34217772
Hailemariam et al. (2013)	0.21614861	0.0788116	0.35348564
Fentaw et al. (2020)	0.1529856	0.07662833	0.22934288
Kahsay et al. (2023)	0.21055301	0.07673436	0.34437165
Combined	0.19943182	0.07997562	0.31888802

### Trim and fill analysis

A trim and fill analysis was performed to evaluate the influence of overlooked studies on the prevalence of *N. gonorrhea* among STI suspected patients in Ethiopia. The inclusion of five additional studies resulted in a shift in the pooled prevalence from 19.9 to 6.2% (95% CI:-6.8–19.3).

### Antimicrobial resistance prevalence of *Neisseria gonorrhea* in Ethiopia

**Figure 7:**
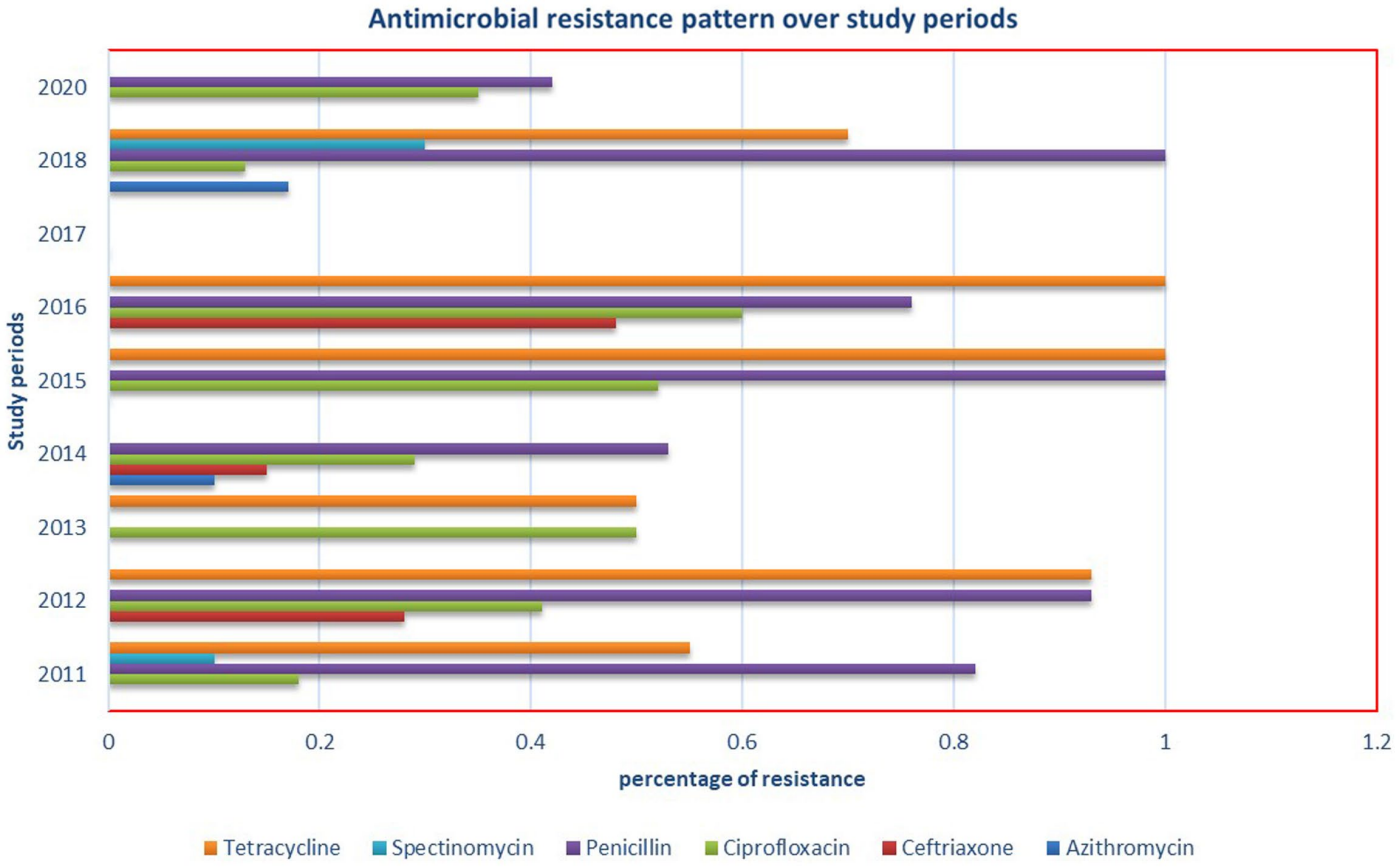
Antimicrobial resistance pattern of *N. gonorrhea* over the study periods in Ethiopia.

We systematically gathered data on the sensitivity of *N. gonorrhea* to six antimicrobials and analyzed the pooled estimate of resistance. Spectinomycin and ceftriaxone demonstrated excellent efficacy against *N. gonorrhea*, while tetracycline and penicillin were found to be the least effective drugs (Table 4). Notably, this meta-analysis revealed a significantly higher prevalence of resistance to ciprofloxacin (37%), a drug currently used for *N. gonorrhea* treatment. Additionally, an investigation into the antimicrobial resistance pattern of *N. gonorrhea* over the study periods did not reveal any discernible pattern (Figure 7).

**Table 4:** Pooled estimates of antimicrobial resistance prevalence of *N. gonorrhea* in Ethiopia.

Antimicrobials	Studies	Total number of isolate	Pooled prevalence of resistance %(95% CI)	I^2^%	*P*-value
Azithromycin	Fentaw et al.				
	Kahsay et al.	384	10 (7–13)	0	0.357
Ceftriaxone	Ali et al.				
	Fentaw et al.				
	Kahsay et al.				
	Sahile et al.				
	Tibebu et al.				
	Yeshanew et al.	490	9 (3–16)	93.9	<0.001
Ciprofloxacin	Ali et al.				
	Fentaw et al.				
	Hailemariam et al.				
	Kahsay et al.				
	Mulu et al.				
	Sahile et al.				
	Tibebu et al.				
	Yeshanew et al.	505	37 (20–54)	89.8	<0.001
Penicillin	Ali et al.				
	Fentaw et al.				
	Hailemariam et al.				
	Kahsay et al.				
	Sahile et al.				
	Tibebu et al.				
	Yeshanew et al.	501	79 (63–94)	98.1	<0.001
Tetracycline	Ali et al.				
	Hailemariam et al.				
	Kahsay et al.				
	Mulu et al.				
	Tibebu et al.				
	Yeshanew et al.	113	93 (87–99)	79.1	<0.001
Spectinomycin	Ali et al.				
	Hailemariam et al.				
	Kahsay et al.				
	Sahile et al.	86	2(−2–5)	69.3	0.021

## Discussion

The objective of this systematic review and meta-analysis was to assess the national pooled prevalence of *N. gonorrhea* among suspected STI patients and evaluate the resistance profile of common antimicrobial agents used against *N. gonorrhea* in Ethiopia. The estimated pooled prevalence of *N. gonorrhea* among STI suspected patients in Ethiopia was found to be 20% (95% CI 8–30, I^2^ = 99.0%; *p* < 0.001). This prevalence diverges significantly from global meta-analyzed reports in 2016, which indicated an *N. gonorrhea* prevalence of 0.9% in adult males and 0.7% in adult females in the general population (Rowley et al., 2019). Recent studies in 2022 revealed lower prevalence rates in developed regions, such as Europe (<0.1%), North America (0.27%), and higher rates in the Western Pacific (7.06%) and Africa (14%) [Benin: 0.9%, Malawi: 9.8%, South Africa: 3.3%] (Whelan et al., 2021). Similarly, a study in Latin America reported a pooled *N. gonorrhea* prevalence of 2.9% in young people (Vallejo-Ortega et al., 2023). While another study found gonorrhea prevalence at 1.46% in Latin America and the Caribbean, rising to 5.68% in high-risk populations (Bardach et al., 2023). In contrast, studies in China, sub-Saharan Africa, and South Africa reported *N. gonorrhea* prevalence rates of 6, 5.46, and 10.1%, respectively (Su et al., 2015; Kularatne et al., 2018; Kassa et al., 2020).

This variation might be due to differences in the study population, as this study focused on STI suspected patients, which might result in an inflation of prevalence compared to the general population, alongside cultural and geographical variations.

Unlike developed nations, there is a lack of proactive response toward monitoring *N. gonorrhea* AMR in Africa. A recent systematic review and meta-analysis across 11 Sub-Saharan countries, including Ethiopia, highlighted substantial resistance to ciprofloxacin, benzylpenicillin, and tetracycline (Whiley et al., 2012). For instance, in Cameroon, approximately 80.1, 64.4, and 58.4% of *N. gonorrhea* strains showed resistance to benzylpenicillin, ciprofloxacin, and tetracycline, respectively (Crucitti et al., 2020). Our study mirrored these findings, indicating the highest prevalence of resistance to tetracycline (97%), penicillin (79%), and ciprofloxacin (37%). The consistency across studies underscores the commonality of *N. gonorrhea* AMR patterns associated with specific antimicrobial classes, emphasizing the urgent need for routine AMR monitoring, antimicrobial consumption surveillance, and improved etiologic diagnostics. It also highlights the necessity for well-organized training for healthcare practitioners, quality assurance for laboratories, and the establishment of centers for regular AMR monitoring.

In contrast to a study conducted in Cameroon, which revealed a modest prevalence of resistance to ceftriaxone (1.8%) and azithromycin (2.1%), our study reported a moderate level of resistance to ceftriaxone (9%) and azithromycin (10%). Noteworthy is our unique finding of macrolide resistance, supported by a study from South Africa in the province of KwaZulu-Natal, which identified azithromycin resistance in 68% of *N. gonorrhea* bacteria, of which 71% exhibited multidrug resistance (Rambaran et al., 2019). Interestingly, a recent systematic and meta-analysis study in Papua New Guinea in 2022 indicated a pooled prevalence estimate of 37.3% for penicillin, 10.3% for tetracycline, and 1.7% for ciprofloxacin resistance (Willie et al., 2022). This finding is notably lower compared to our study. Spectinomycin emerged as the least resistant drug in systematic and meta-analysis studies conducted in sub-Saharan Africa (Yakobi and Pooe, 2022), Cameroon (Crucitti et al., 2020), and Papua New Guinea (Willie et al., 2022) spectinomycin is the least resistant drug reported. The discrepancies observed could potentially be attributed to differences in sample sizes of isolates tested for *N. gonorrhea* surveillance and monitoring,

AMR surveillance, laboratory setups, rapid point-of-care tests for the detection of *N. gonorrhea*, and the technical capabilities of laboratory technicians.

The subgroup analysis based on different regions of Ethiopia provided valuable insights into the varied prevalence of *N. gonorrhea* among STI suspected patients. Notably, the highest pooled prevalence of 55% in Addis Ababa and the subsequent prevalence of 16% in the Amhara region underscore the regional disparities in the burden of *N. gonorrhea*. Conversely, the notably lower prevalence of 4% in the Southern Nations, Nationalities, and Peoples’ Region (SNNPR) highlights potential regional variations in STI prevalence and emphasizes the need for targeted interventions.

Interestingly, the subgroup analysis conducted based on the study periods did not reveal a significant difference in the pooled prevalence of *N. gonorrhea*. This suggests a relatively consistent prevalence over time, highlighting the persistent nature of the issue and the need for continuous monitoring and intervention efforts.

The trim and fill analysis addressed the potential impact of overlooked studies on the prevalence estimate. The inclusion of five additional studies resulted in a substantial shift in the pooled prevalence from 19.9 to 6.2%, emphasizing the significance of considering potential publication bias and the importance of a more nuanced interpretation of the prevalence estimate.

Our sensitivity analysis using a random effects model demonstrated the robustness of the prevalence estimate, as the exclusion of each study did not significantly alter the overall prevalence. This lends credibility to our findings and reinforces the stability of the estimated prevalence of *N. gonorrhea* among STI suspected patients in Ethiopia.

The assessment of publication bias through a subjective examination of the funnel plot and Egger’s regression test revealed a significant presence of bias (*p*-value of 0.001). The subsequent trim and fill analysis, which added five studies, further supported the indication of potentially overlooked small studies contributing to this bias. This underscores the importance of interpreting the prevalence estimate cautiously and considering potential sources of bias in the analysis.

The observed substantial heterogeneity among the included studies, as indicated by I^2^ = 98.1%, remained a challenge. Despite attempts with a weighted inverse variance random-effects model, the heterogeneity persisted. The subjective assessment through a forest plot and subgroup analysis provided insights, but the ultimate meta-regression using sample size revealed the absence of significant heterogeneity. This finding suggests that other unexplored factors may contribute to the observed variability between studies.

## Conclusion

Our exhaustive systematic review and meta-analysis focused on *N. gonorrhea* in Ethiopia, specifically examining its prevalence among STI suspected patients and its antimicrobial susceptibility. The findings provide valuable insights, indicating a pooled prevalence of 20% for *N. gonorrhea* among STI suspected patients. Notably, the bacterium exhibited significant resistance to penicillin and tetracycline, with a particularly alarming high resistance rate to ciprofloxacin. However, ceftriaxone, azithromycin, and spectinomycin emerged as effective alternative treatments in the face of antimicrobial resistance.

## Strengths and limitations of the study

While our study on the prevalence of *N. gonorrhea* among STI suspected patients and its antimicrobial resistance in Ethiopia provides valuable insights, it is essential to acknowledge certain limitations for a nuanced interpretation. Notably, there is significant heterogeneity among the included studies, attributed to variations in sample size and study designs. This diversity necessitates caution when generalizing the pooled prevalence estimate, as potential biases may exist. Another notable limitation is the prevalent use of the disk diffusion method over the recommended minimum inhibitory concentration (MIC) method in primary studies. Despite these limitations, our study holds notable strengths. It offers a comprehensive analysis of *N. gonorrhea* prevalence and antimicrobial resistance in Ethiopia, providing valuable insights into the current landscape. The inclusion of a diverse range of studies enhances the robustness of our findings, and the meta-analysis approach allows for a comprehensive overview of the available data. These strengths contribute to the overall significance and reliability of our study’s findings.

## Data availability statement

The original contributions presented in the study are included in the article/supplementary material, further inquiries can be directed to the corresponding author.

## Author contributions

MG: Conceptualization, Formal analysis, Methodology, Writing – original draft, Writing – review & editing. NT: Conceptualization, Data curation, Formal analysis, Writing – original draft, Writing – review & editing. TS: Investigation, Methodology, Writing – original draft, Writing – review & editing. MD: Investigation, Validation, Writing – original draft, Writing – review & editing. TK: Conceptualization, Methodology, Writing – original draft, Writing – review & editing. TW: Investigation, Methodology, Writing – original draft, Writing – review & editing. EA: Formal analysis, Investigation, Methodology, Writing – original draft, Writing – review & editing. MH: Conceptualization, Formal analysis, Methodology, Writing – original draft, Writing – review & editing.
